# Solvent Effect on Electrochemical CO_2_ Reduction Reaction on Nanostructured Copper Electrodes

**DOI:** 10.1021/acs.jpcc.3c03257

**Published:** 2023-07-12

**Authors:** Connor Deacon-Price, Alisson H. M. da Silva, Cássia S. Santana, Marc T. M. Koper, Amanda C. Garcia

**Affiliations:** † Van’t Hoff Institute for Molecular Sciences, University of Amsterdam, Science Park 904, 1098 XH, Amsterdam, The Netherlands; ‡ Leiden Institute of Chemistry, Leiden University, Gorlaeus Laboratories, P.O. Box 9502, 2300 RA, Leiden, The Netherlands

## Abstract

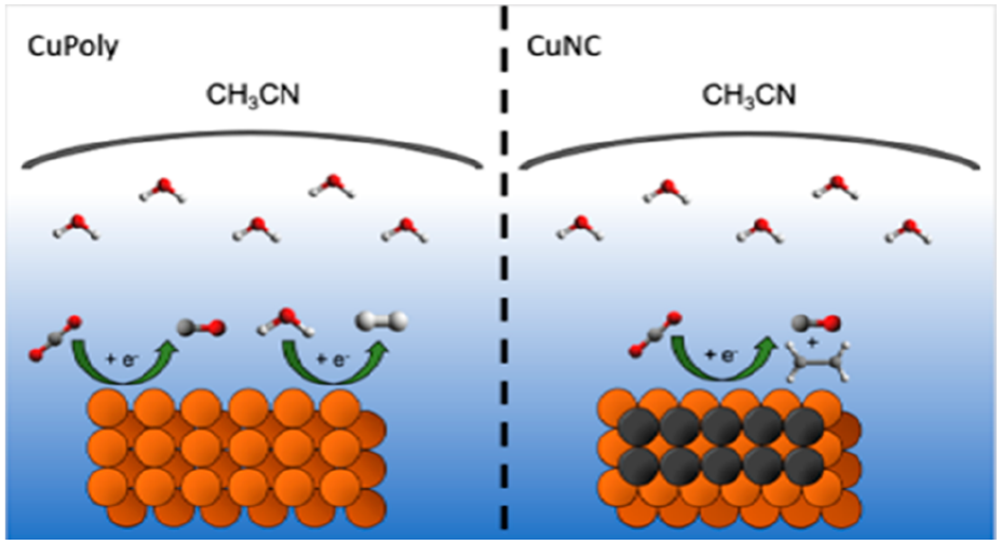

The electrochemical reduction of CO_2_ (CO_2_RR) is a sustainable alternative for producing fuels and chemicals, although the production of highly desired hydrocarbons is still a challenge due to the higher overpotential requirement in combination with the competitive hydrogen evolution reaction (HER). Tailoring the electrolyte composition is a possible strategy to favor the CO_2_RR over the HER. In this work we studied the solvent effect on the CO_2_RR on a nanostructured Cu electrode in acetonitrile solvent with different amounts of water. Similar to what has been observed for aqueous media, our online gas chromatography results showed that CO_2_RR in acetonitrile solvent is also structure-dependent, since nanocube-covered copper (CuNC) was the only surface (in comparison to polycrystalline Cu) capable of producing a detectable amount of ethylene (10% FE), provided there is enough water present in the electrolyte (>500 mM). In situ Fourier Transform Infrared (FTIR) spectroscopy showed that in acetonitrile solvent the presence of CO_2_ strongly inhibits HER by driving away water from the interface. CO is by far the main product of CO_2_RR in acetonitrile (>85% Faradaic efficiency), but adsorbed CO is not detected. This suggests that in acetonitrile media CO adsorption is inhibited compared to aqueous media. Remarkably, the addition of water to acetonitrile has little quantitative and almost no qualitative effect on the activity and selectivity of the CO_2_RR. This indicates that water is not strongly involved in the rate-determining step of the CO_2_RR in acetonitrile. Only at the highest water concentrations and at the CuNC surface, the CO coverage becomes high enough that a small amount of C_2+_ product is formed.

## 1 Introduction

The electrochemical reduction of carbon dioxide (CO_2_RR) offers a sustainable pathway to address some of the future energy needs by converting waste into fuels and chemicals without relying on fossil fuels derivatives.^1−4^ Although electrochemistry has the promising ability to operate at room temperature and pressure,^5^ CO_2_RR is still hampered by low energy efficiency related to the high overpotential required, especially in the conversion of CO_2_ to highly reduced C_2_^+^ products,^6^ and the poor long-term stability of electrocatalysts.^7^ Furthermore, because most CO_2_ electrolyses are performed in aqueous media, hydrogen evolution reaction (HER) occurs simultaneously with CO_2_RR, limiting the Faradaic efficiency (FE) toward CO_2_RR products.^8,9^

Tailoring the catalyst surface is the most common way to steer activity and selectivity to favor CO_2_RR over HER.^10,11^ Many recent studies, however, have showed the importance of the electrolyte composition on the reaction rate of CO_2_ reduction and H_2_ evolution.^12−15^ Recently, Monteiro et al.^16^ showed that the electrochemical reduction of CO_2_ to CO on gold, copper, and silver electrodes requires the presence of a cation to coordinate and, hence, to stabilize the first electron-transfer intermediate CO_2_^–^. Nevertheless, an intermediate cation concentration is preferable, as cations also promote the concomitant water and bicarbonate reduction, favoring hydrogen evolution.^2,12,15,17−20^ Therefore, if water is the main proton donor, the activity for the hydrogen evolution reaction can be altered by changing the cation and its interfacial concentration.

An alternative strategy to promote CO_2_RR over HER is to replace water with an organic solvent, such as acetonitrile,^21^ dimethylformamide,^22^ or methanol.^23^ These solvents have lower proton and/or water availability, which should lower the hydrogen evolution rate. Previous studies by Amatore and Saveánt^22^ showed that CO_2_RR in dry dimethylformamide proceeds through three competing pathways: (1) oxalate formation through self-coupling of the CO_2_^•–^ anion radicals, (2) CO and CO_3_^2–^ formation via oxygen–carbon coupling of CO_2_^•–^ with CO_2_, and (3) formate formation through protonation of CO_2_^•–^ by residual water, followed by a homogeneous electron transfer from CO_2_^•–^. On the other hand, Figueiredo et al.,^21^ using a copper electrode in acetonitrile containing small amounts of water, found that the main products from CO_2_RR are carbonate, bicarbonate, and CO. However, the formation of CO and carbonate was suggested to be decoupled from each other by the influence of residual water. The formation of carbonate and bicarbonate species appears to be the result of a solution-phase reaction of CO_2_ with electrochemically generated OH^–^ from the water reduction.

Considering the key role of the solvent composition on CO_2_ reduction, it would be attractive to understand the mechanism of the CO_2_RR toward hydrocarbon products in acetonitrile solvent and how the presence of water influences the product distribution and the competition between the CO_2_RR and HER.

Much of the work present in the literature attempting to understand the CO_2_RR mechanism in aqueous electrolytes has been based on theoretical and experimental studies with well-oriented Cu single crystal surfaces. These studies showed that Cu(100) surfaces^24−28^ are particularly interesting because of their high selectivity to C_2+_ products, like ethylene and ethanol^21,24,29,30^ in comparison to Cu(111) surfaces, which are more selective for the generation of CH_4_.^24,25,27,28^ The higher selectivity of Cu(100) toward hydrocarbons compared to Cu(111) was previously related to the CO-binding energy on the electrode surface,^25,31−33^ which is considered to be crucial for the OC–CO dimerization and, therefore, to make hydrocarbon products.^27,34−36^ More recently, studies using ultrahigh vacuum preparation and spectroscopic characterization,^29^ however, showed that copper single crystals with clean, flat, and atomically ordered surfaces do not exhibit high selectivity to hydrocarbons, instead favoring hydrogen evolution. Only the introduction of defects and roughness, for example, by electropolishing or O_2_-plasma treatment, shifts the selectivity toward hydrocarbons. This change in mechanism is due to the stronger CO-binding strength on the defected surface, which is more predominant on Cu_2_O species that originated on the surface of Cu(100) after the electrochemical pretreatment.

In this work, we prepared copper oxide-derived (Cu_*x*_O) electrodes with different morphologies through electro-oxidative/reductive cycles and tested their activity and selectivity for the CO_2_RR in acetonitrile solution. Through a combination of electrochemical measurements with online gas chromatography (GC) and in situ Fourier Transform Infrared (FTIR) spectroscopy, we studied the electrochemical reduction of CO_2_ in acetonitrile solvent in the presence of different amounts of water on Cu nanocubes (CuNC) and polycrystalline Cu (CuPoly) electrodes. Our results show that, in acetonitrile, CO is by far the dominant product, regardless of the water concentration in the electrolyte. This shows that the competition between the CO_2_RR and HER in acetonitrile is very different from the situation in aqueous media. However, similar to aqueous media,^27,37^ CO_2_RR selectivity toward C_2+_ products in acetonitrile is dependent on the electrode structure and electrolyte composition. CuNC is the only surface capable of producing a detectable amount of ethylene (10% FE), provided there is enough water present in the electrolyte (>500 mM).

## 2 Materials and Methods

### 2.1 Cleaning and Sample Preparation

All water used in this work (resistivity >18.2 MΩ·cm, TOC < 5 ppb) was purified with a Millipore Milli-Q system. All glassware was cleaned from organic contamination by soaking overnight in an aqueous solution of 1 g L^–1^ of KMnO_4_ (Fluka, ACS reagent) and 0.5 M KOH (Sigma-Aldrich, ACS reagent). Before experiments, this solution was drained, and residual MnO_4_^–^ was decomposed by immersing the glassware in dilute piranha solution (3:1 v/v mix of H_2_SO_4_ (Sigma-Aldrich, 95–98%)/H_2_O_2_ (Sigma-Aldrich, 30% w/w)). It was subsequently drained, and all the glassware was boiled in water at least three times to remove inorganic contaminants.

KHCO_3_ (Sigma-Aldrich, 99.95%), KCl (Sigma-Aldrich, >99%), HClO_4_ (Sigma-Aldrich, 60%), CH_3_CN (Biosolve, 99.9%), and C_8_H_20_ClN (TEACl; ThermoFisher Scientific, 99%) were used as received. CO_2_ (Linde, 4.5 purity) and Ar (Linde, 6.0 purity) gases were used to saturate and deaerate the electrolyte solution, respectively.

To ensure that the solvent was as water-free as possible, prior to the experiments, acetonitrile was dried over 3 Å molecular sieves, which had been previously stored at 130 °C. Molecular sieves were first added to a Schlenk tube, which was then flame-dried under vacuum to remove water vapor. A positive pressure of N_2_ was applied to the vessel in which acetonitrile was stored. The solvent was then stored under N_2_ for a minimum of 24 h before use. Karl Fischer titration showed 1.6 mM (±0.5 mM) H_2_O content before use.

### 2.2 Electrochemical Measurements

Electrochemical measurements were carried out in a single-compartment three-electrode cell with a MultiAutoLab M101 Potentiostat (Metrohm). A polycrystalline Cu disk (CuPoly – ϕ 5 mm) or Cu_*x*_O was used as the working electrode0 (WE). Prior to experiments, the CuPoly disk was polished with a 5.0 μm diamond suspension (Buehler), then rinsed with ultrapure water, followed by sonication in ultrapure water. Next, the electrode was electropolished in a 66% H_3_PO_4_ (Fisher Scientific, 85%) aqueous solution. A Pt wire was used as the counter electrode (CE) and Ag/AgCl/KCl_sat_ or commercial leak-free Ag/Ag^+^ (Alvatek) was used as the reference electrode (RE) in aqueous and organic solvents, respectively. Prior to experiments, we tested the possibility of shifting the potential of the reference Ag/Ag^+^ electrode due to the addition of H_2_O or saturation with CO_2_ by adding the ferrocene/ferrocenium (Fc/Fc^+^) redox couple in solution. As shown in Figure S1, no shift was observed.

All potentials measured against the Ag/AgCl/KCl_sat_ electrode were converted to their reversible hydrogen electrode (RHE) values according to eq 1:^12^
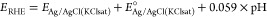
1

All the potential values (*E*) were corrected for ohmic drop according to eq 2:^14^
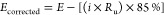
2where *R*_u_ is the uncompensated resistance measured (ca. 400 Ω in organic solvent). Electrolysis measurements were carried out at −2.0 V versus Ag/Ag^+^. The experiments were performed using a homemade two compartment (H-type) electrochemical cell.^38^ An anionic exchange membrane (AHO, AGC Inc.) was used to separate the cathodic and anodic compartments, which were filled with 10 mL of 0.1 M TEACl in MeCN solvent. In the cathodic compartment, CO_2_ was continuously bubbled at a fixed flow rate of 15 mL min^–1^ from directly below the WE to avoid mass transport limitations. Different amounts of ultrapure water were added (10, 100, 500, and 1000 mM) into the acetonitrile electrolyte to assess the effect of the proton donor content. A dimensionally stable anode (DSA, Magneto) counter electrode (CE) and a commercial leak-free Ag/Ag^+^ (Alvatek) reference electrode (RE) were used for all electrolysis experiments. Water concentrations were measured using a Karl Fischer titration, finding no significant change before and after bubbling of CO_2_ into the electrolyte. The potential of the working electrode was controlled by a Bio-Logic potentiostat/galvanostat/EIS (SP-300). Gaseous products were analyzed online using a Shimadzu 2014 gas chromatograph (GC) with two detectors (one TCD and one FID). The analyte was separated before analysis by FID via an RTX-1 column and for TCD via a Shincarbon column. The column temperature was held at 40 °C for 3 min before being increased to 130 °C at a heating rate of 150 °C min^–1^. Liquid products were measured by high performance liquid chromatography (Prominence HPLC, Shimadzu). A 5 mM H_2_SO_4_ solution was used as the eluent, separating the analyte over an Aminex HPX-87H (Biorad) column. Flow rate was maintained at 0.6 mL min^–1^ at a column temperature of 45 °C.

### 2.3 In Situ Fourier Transform Infrared Spectroscopy

To provide information about the surface-adsorbed intermediates and products of the electrochemical reduction of carbon dioxide, in situ FTIR spectra were collected. The FTIR instrument was a Bruker Vertex 80 V IR spectrometer equipped with a liquid nitrogen cooled detector. In situ FTIR experiments were performed in a three electrode spectro-electrochemical cell with a CaF_2_ prism attached to the bottom of the cell. Details concerning the cell are described in the literature.^39^ FTIR spectra were obtained in the wavenumber range between 4000 and 900 cm^–1^. Spectra were collected in 0.1 V steps from −1.0 to −2.5 V vs RHE under an argon or CO_2_ atmosphere. They were computed from the average of 100 interferograms with the spectral resolution set to 8 cm^–1^. Spectra are presented as reflectance, according to *A* = −log (*R*/*R*_0_), where *R* and *R*_0_ are the reflectances corresponding to the single beam spectra obtained at the sample and reference potentials, respectively. In these difference spectra, negative bands (pointing down) correspond to the species that were present on or near the electrode surface at the reference potential and that are “consumed” at the sample potential. Positive bands (pointing up) correspond to the formation of species at the sample potential. All the spectro-electrochemical experiments were performed at room temperature, with Ag/Ag^+^ and platinum coils used as reference and counter electrodes, respectively.

### 2.4 Cu_*x*_O-Derived Nanoparticle Synthesis

Shape-controlled nanoparticle (NP) synthesis was performed via the procedure previously described by Roberts et al.^40^ A CuPoly plate (WE) was placed in a three-electrode cell (Pt wire, CE; Ag/AgCl/KCl_sat._, RE) and then subjected to four oxidation–reduction potential cycles. The electrodes were directly submerged in an aqueous electrolyte solution of 0.1 M KHCO_3_ solution +4 mM KCl purged with CO_2_ to maintain a pH of 6.8.^40^ Potentials were cycled from 0.0 V to *E*_upper_ to −1.2 V versus RHE, where *E*_upper_ = 0.7, 0.9, or 1.1 V versus RHE, at a scan rate of 5 mV s^–1^.^40^

The relative electrochemically active surface area (ECSA) of the different copper electrodes were determined by the procedure previously described by Kanan et al.^30,41,42^ Argon was bubbled through a 0.1 M HClO_4_ solution in a three-electrode cell configuration for 20 min to remove all of the dissolved oxygen. To prevent the redissolution of ambient oxygen, a constant flow of Ar was maintained over the solution. Cyclic voltammetry (CV) was performed in the double layer (DL) region, corresponding to a potential range of 0.00 V to −0.12 V vs RHE. After repeating the measurements over six different scan rates (10, 20, 40, 60, 80, and 100 mV s^–1^), the DL capacitance was extracted from the slope of the linear plot of DL current against scan rate. The positive current was measured at −0.05 V, where no electrochemical activity was found to take place. The roughness factor was then calculated, since the DL capacitance value in the DL region is expected to be proportional to the electrochemically active surface area. By comparison with CuPoly, the RF of the CuNPs can be defined by normalizing the measured capacitances with respect to that of CuPoly. CuPoly is set as reference, which is defined as RF = 1.

For each experimental condition, we used freshly prepared electrodes, with their structures being verified in 0.1 M NaOH before their use.

### 2.5 Physico-Chemical Characterization

The morphology of the synthesized Cu_*x*_O-based nanoparticles was analyzed by using a Scanning Electron Microscopy (SEM) technique Apreo SEM (ThermoFisher Scientific) with an acceleration voltage of 15 kV and an electron beam current of 0.4 nA.

## 3 Results

### 3.1 Physical and Electrochemical Characterization of Oxide-Derived Copper Electrodes

**Figure 1:**
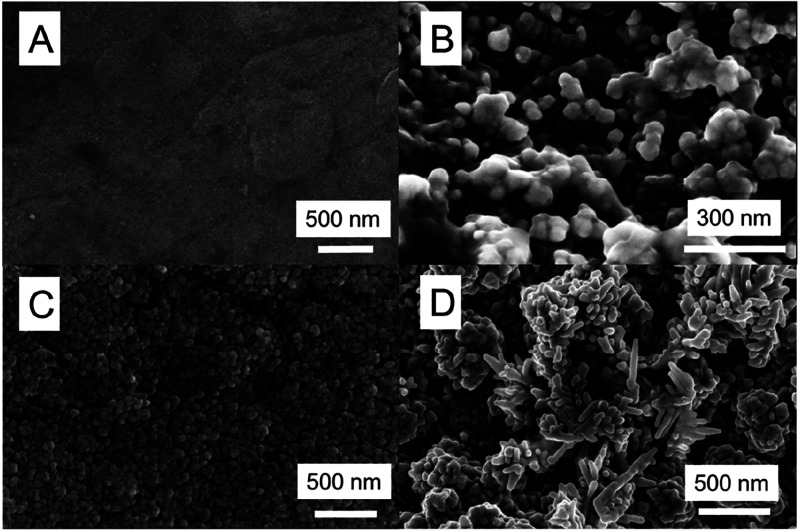
Scanning electron microscopy images of CuPoly(A), CuNS (B), CuNC (C), and CuND (D) oxide-derived materials.

SEM images revealed that the electrochemical oxidation–reduction treatment of polycrystalline Cu (CuPoly) in the Cl^–^ solution results in different nanostructures, as shown in Figure 1, in agreement with the literature.^40,43^ CuPoly (Figure 1A) is included for comparison, and the surface of which remains featureless at the nanoscale level. By sweeping the electrode potential from 0.0 to 0.7 V versus RHE, nanoparticles with the shape of irregular spheres of ∼50 nm were formed (CuNS, Figure 1B), whereas by sweeping the potential up to 0.9 and 1.1 V, irregular nanocubes of ∼70 nm (CuNC, Figure 1C) and nanodendrites of ∼300 nm (CuND, Figure 1D) were obtained, respectively. These materials are referred to as oxide-derived Cu electrodes (Cu_*x*_O), as they are metallic electrodes resulting from the reduction of an oxide.^43^ They are composed of mostly metallic copper (Cu^0^) and copper oxide (Cu(I)), with a higher proportion of the latter being present than in CuPoly.^40,44^

**Figure 2:**
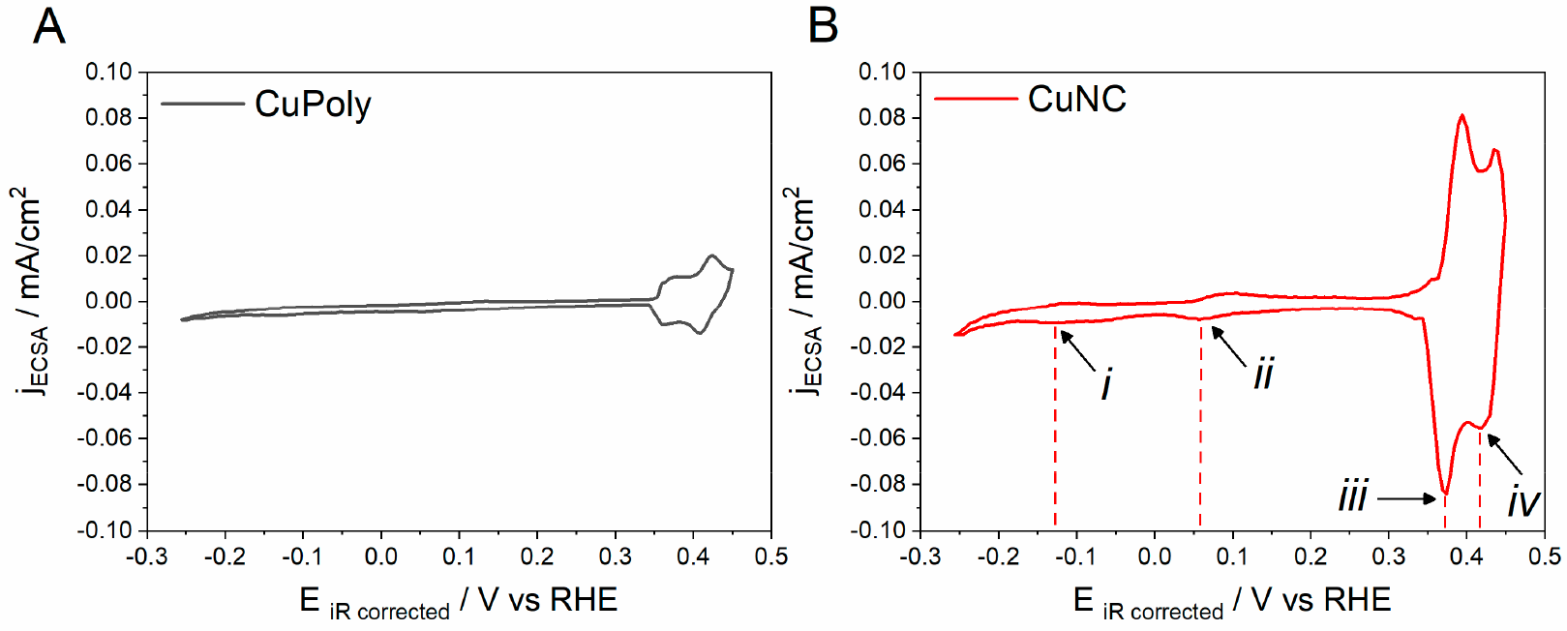
Cyclic voltammograms of Cu polycrystalline (CuPoly; A) and Cu nanocube (CuNC; B) electrodes in 0.1 M NaOH, under an Ar atmosphere, at a scan rate of 50 mV s^–1^.

The synthesized Cu_*x*_O-derived electrode surfaces were also characterized electrochemically by performing cyclic voltammetry (CV) in a blank electrolyte (0.1 M NaOH) between −0.25 and 0.45 V versus RHE. The obtained profiles for CuPoly and CuNC are shown in Figure 2, whereas CuNS and CuND profiles are depicted in Figure S2.

For all Cu surfaces, multiple well-defined peaks are observed in the potential range between −0.25 V < *E* < 0.45 V versus RHE, and the double layer for all electrodes are similar, in agreement with the literature for metallic copper.^45^ A peak at −0.13 V (Figure 2B,*i*) is observed on the CuNC (Figure 2B) and CuND (Figure S2) electrodes, and it corresponds to the adsorption/desorption of OH^–^ species on (100) terraces, providing evidence for the increased exposure of this facet.^17,46^ The peak at 0.06 V (Figure 2B,*ii*) corresponds to the adsorption/desorption of OH^–^ on the (111) terraces.

The presence of defects or low coordinate sites are evidenced by the small voltammetric feature at 0.30 V versus RHE,^47,48^ appearing before the prominent features centered at 0.37 V (Figure 2B, *iii*) and 0.42 V (Figure 2B, *iv*), which are due to the redox activity of Cu to Cu_2_O over different domains of the copper surface.^45^ For Cu(100) crystallographic orientation, the same peaks are attributed to the oxidation of (100) domains,^48^ which agrees with the fact that CuNC (Figure 2B) has more well-defined peaks in this potential region.

As Cu electrodes with (100) crystallographic orientation are well-known to possess a high selectivity toward the C–C coupling reaction for the CO_2_RR,^44,45,47,49,50^ all subsequent results are related only to CuNC in comparison to CuPoly.

### 3.2 Electrochemical CO_2_ Reduction

#### 3.2.1 Effect of the Solvent

To better understand how electrode structure and electrolyte composition affect both product distribution and the competition between CO_2_RR and HER on the Cu electrode, we studied these reactions in aqueous (0.1 M KHCO_3(aq)_, ∼55 M of H_2_O) and organic (0.1 M TEACl in CH_3_CN) electrolytes through cyclic voltammetry (CV) using CuPoly and CuNC electrodes.

**Figure 3:**
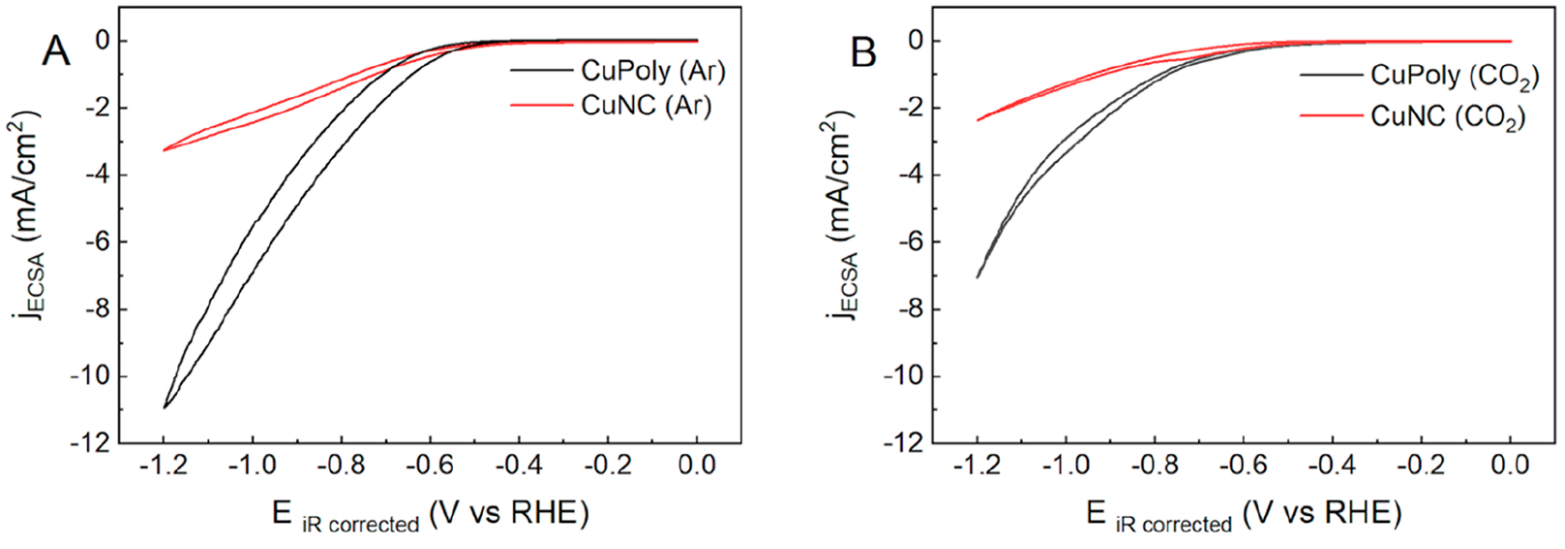
Cyclic voltammograms of CuPoly and CuNC under Ar (A) and CO_2_ (B) atmospheres in 0.1 M KHCO_3(aq)_ at a scan rate of 50 mV s^–1^.

Figure 3 compares the CVs for CuPoly(black line) and CuNC (red line) in an aqueous solution. Hydrogen evolution was observed in an argon-purged electrolyte, as shown in Figure 3A, where an increase in cathodic current density due to water reduction is observed at around −0.55 V versus RHE, for both electrodes.

We also performed cyclic voltammetry in a CO_2_ atmosphere (Figure 3B) in order to distinguish the currents from HER and CO_2_RR. In aqueous solution, a slightly lower reduction current density is observed on both CuPoly and CuNC electrodes, compared to the Ar-saturated electrolyte, suggesting the strong competition between the CO_2_RR and HER.

In both Ar and CO_2_ electrochemical conditions, the CuPoly electrode showed a 4-fold higher current density than CuNC. This difference is at least partially associated with a lower partial current density for HER for CuNC electrode.^29^ Our findings agree with previous work in which Cu(110), which is closer to the copper polycrystalline surface, was shown to be more active for hydrogen evolution than Cu(100).^51^

**Figure 4:**
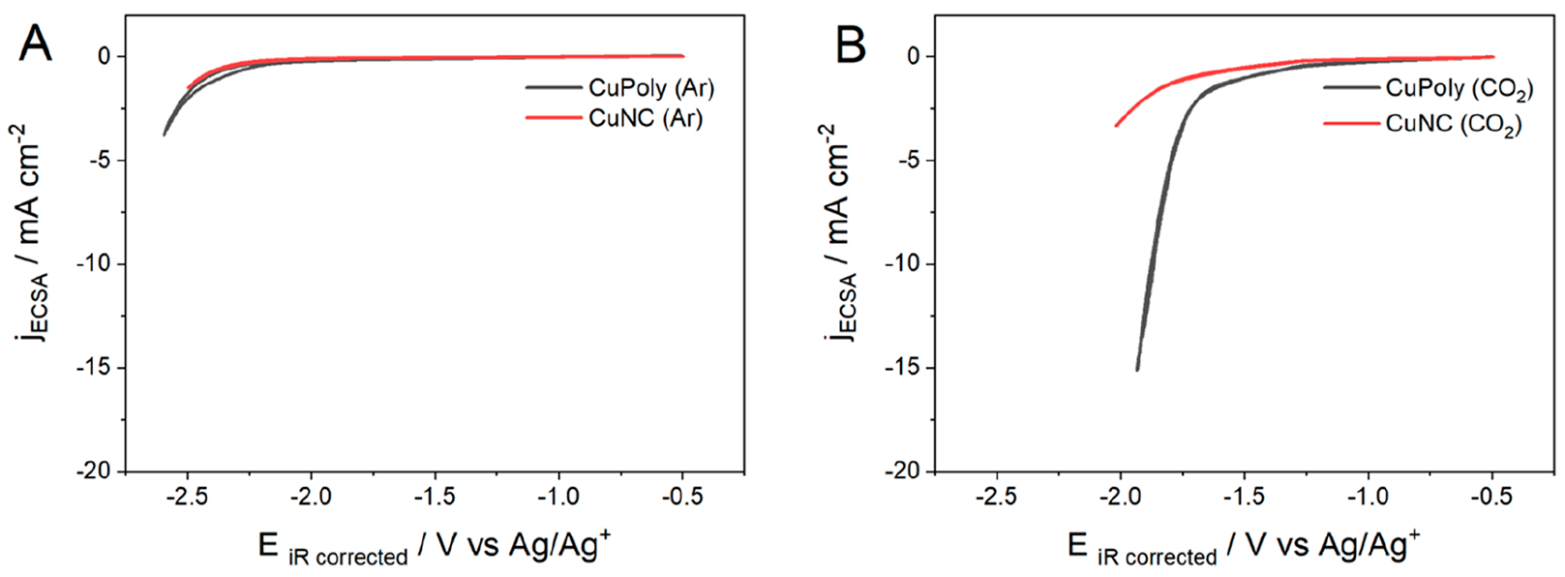
Cyclic voltammograms of CuPoly and CuNC electrodes in MeCN (0.1 M TEACl) under Ar (A) and CO_2_ (B) atmospheres at a scan rate of 50 mV s^–1^.

As mentioned, we also studied the CO_2_RR in acetonitrile solvent to minimize its competition with HER. Figure 4A compares the CVs of both CuPoly and CuNC electrodes in an argon-purged acetonitrile solvent. On both electrodes, an increase in current density is observed only at potentials more negative than −2.0 V versus Ag/Ag^+^. Such increase in current is most likely due to the reduction of residual water present in the acetonitrile electrolyte solution.

Unlike in an aqueous electrolyte, where the competition between the CO_2_RR and HER on Cu often leads to a lower overall current compared to a CO_2_-free electrolyte, in acetonitrile, the CO_2_RR (Figure 4B) leads to higher reduction currents than in Ar-saturated electrolyte (Figure 4A), suggesting the absence of strong competition between both reactions due to low HER activity and to a higher solubility of CO_2_ in acetonitrile compared to water.

The slightly higher current densities observed in Figure 4B in comparison with aqueous electrolytes (Figure 3B), especially on CuPoly (black line), suggest that, in acetonitrile solvent, CO appears to desorb more easily from the electrode surface. Such an observation will be reinforced with the online gas chromatography analysis and in situ FTIR spectroscopy results, to be further discussed below. As in aqueous electrolyte, CuPoly shows a higher CO_2_RR activity than CuNC, although it is not clear if this is for the same reason.

#### 3.2.2 Effect of the Water Content on Activity and Selectivity

**Figure 5:**
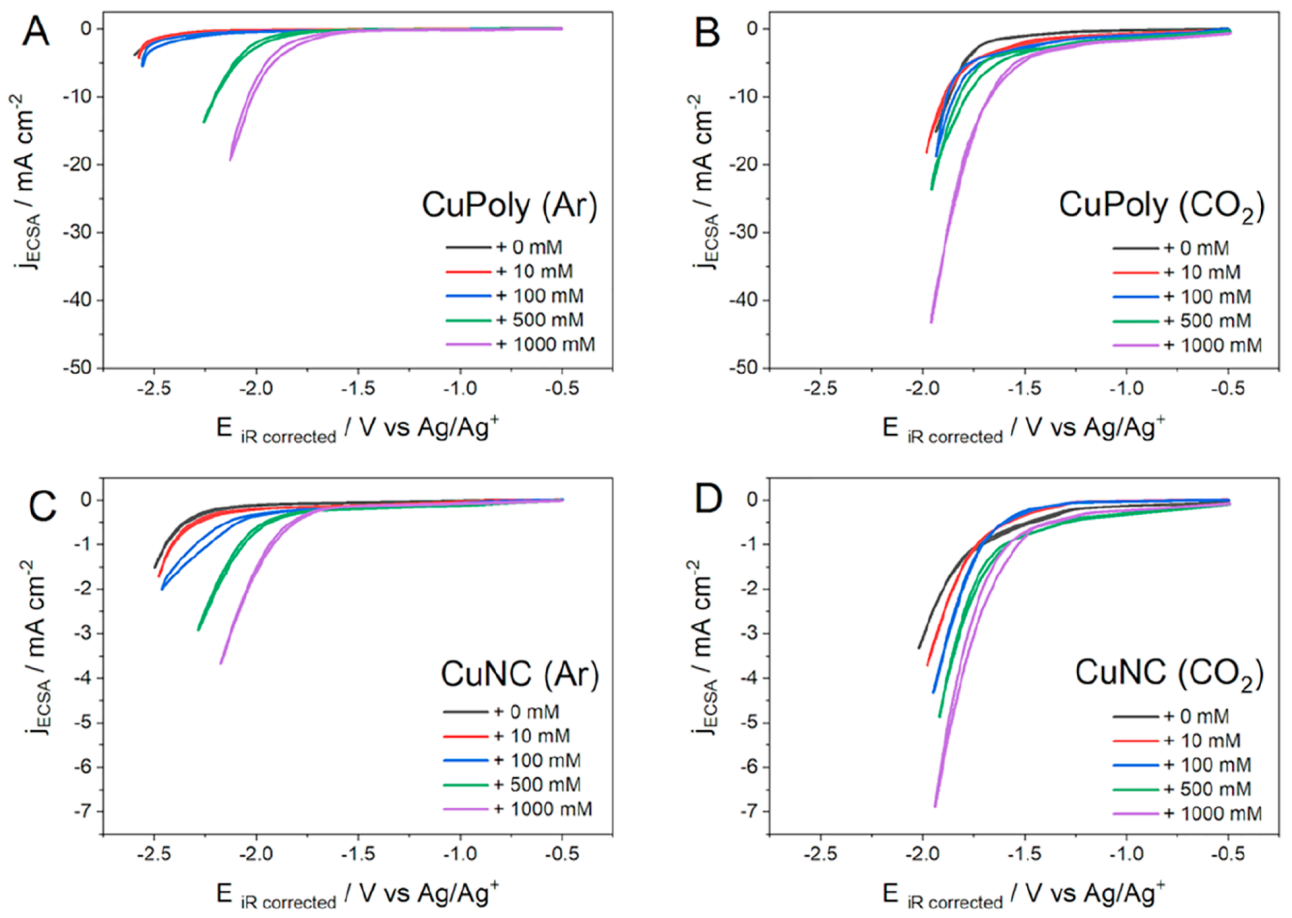
Cyclic voltammograms for CuPoly and CuNC under Ar (A, C) and CO_2_ (B, D), respectively, in acetonitrile (0.1 M TEACl). Scan rate: 50 mV s^–1^.

We further studied the role of water during HER and CO_2_RR in acetonitrile by varying its concentration in the electrolyte. Figure 5 shows the cyclic voltammograms obtained for HER and the CO_2_RR on CuPoly and CuNC in acetonitrile with different water concentrations. The results show that independent of the Cu surface, the HER (Figure 5A,C) is more sensitive to the water content than the CO_2_RR (Figure 5B,D). Moreover, the results in Figure 5 suggest that water is not directly involved in the rate-determining step of CO_2_ reduction.

**Figure 6:**
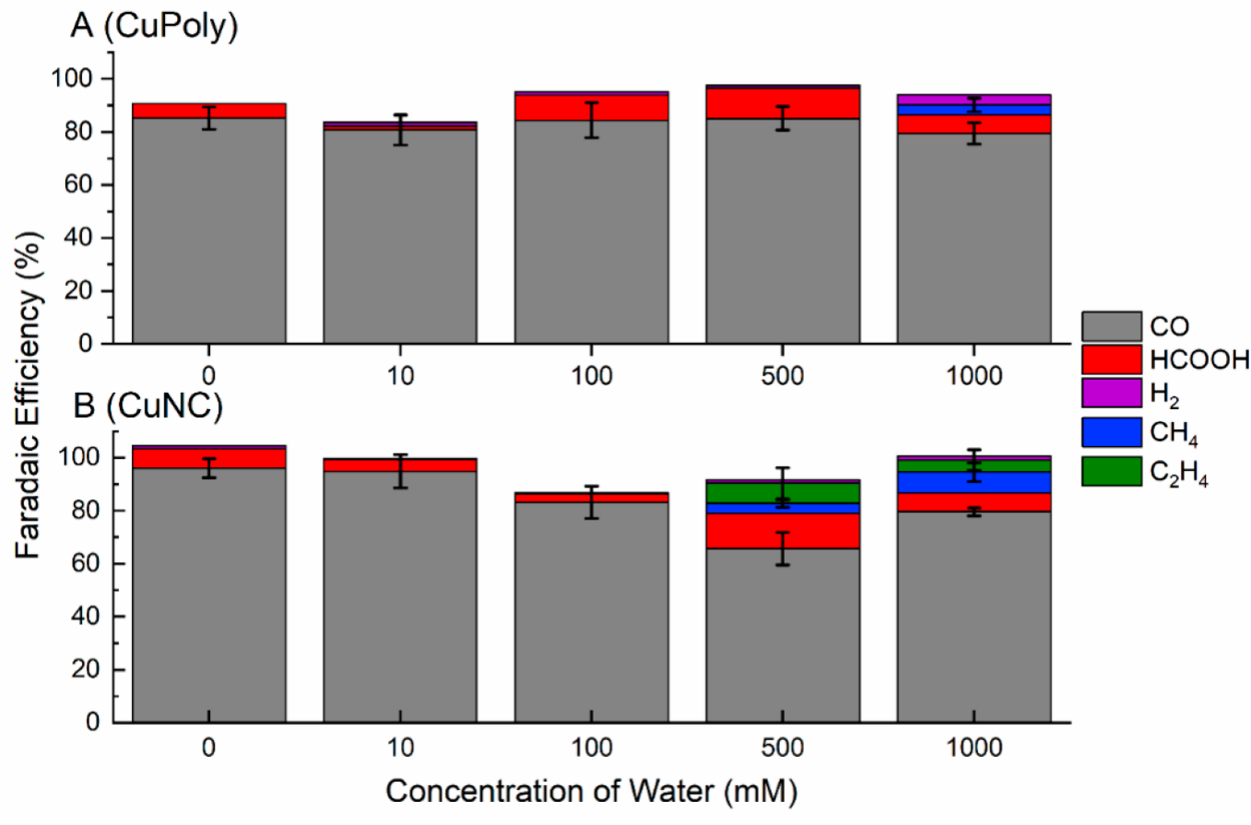
Faradaic efficiencies of CuPoly(A) and CuNC (B) at varying concentrations of water, determined by online gas chromatography at −2.0 V vs Ag/Ag^+^ for 90 min.

To probe the effect of water on the product distribution during the CO_2_RR in acetonitrile solvent, chronoamperometry combined with online GC was carried out with different water concentrations on both CuPoly and CuNC electrodes. Product distributions and the corresponding Faradaic Efficiencies (FE) are depicted in Figure 6, whereas the partial currents for the related products are presented in Figure S3, which confirms higher currents for CuNC than for CuPoly.

The results in Figure 6 show that on both CuPoly and CuNC electrodes, CO is the major product from the CO_2_ reduction in acetonitrile, regardless of the water concentration. This observation is in line with the small effect of the water concentration on the activity of CO_2_RR (Figure 5B,D). Similar to the findings in aqueous media for Cu(100) and Cu-oxide derived in aqueous media,^25,29,43,52−54^ CuNC was the only surface capable of promoting C–C coupling during CO_2_RR in acetonitrile toward ethylene (C_2_H_4_), but only if enough water has been added (>500 mM) to the electrolyte. FE often does not reach 100%, which is thought to be due to a small contribution of carbonate formation.

Interestingly, the online GC analysis shows that the presence of CO_2_ suppresses hydrogen evolution because little to no H_2_ is detected, even at higher amounts of water, where the HER current in the absence of CO_2_ is in fact quite substantial (see the “+1000 mM” curves in Figure 5A,C). Only on CuPoly with 1000 mM H_2_O, there is a measurable FE for hydrogen, but it is still not higher than a few percent. Like in aqueous electrolytes, CuPoly is more active for HER than CuNC.

By comparison, experiments in bicarbonate solution using very similar copper polycrystalline and Cu nanocube electrodes showed a FE for H_2_ of around 75% and 50%, respectively, during CO_2_ reduction.^43^

#### 3.2.3 In Situ FTIR Analysis

To better understand the effect of the water content on the competition between hydrogen evolution and CO_2_ reduction, the reactions in acetonitrile were also investigated by in situ FTIR spectroscopic studies on the CuPoly electrode.

**Figure 7:**
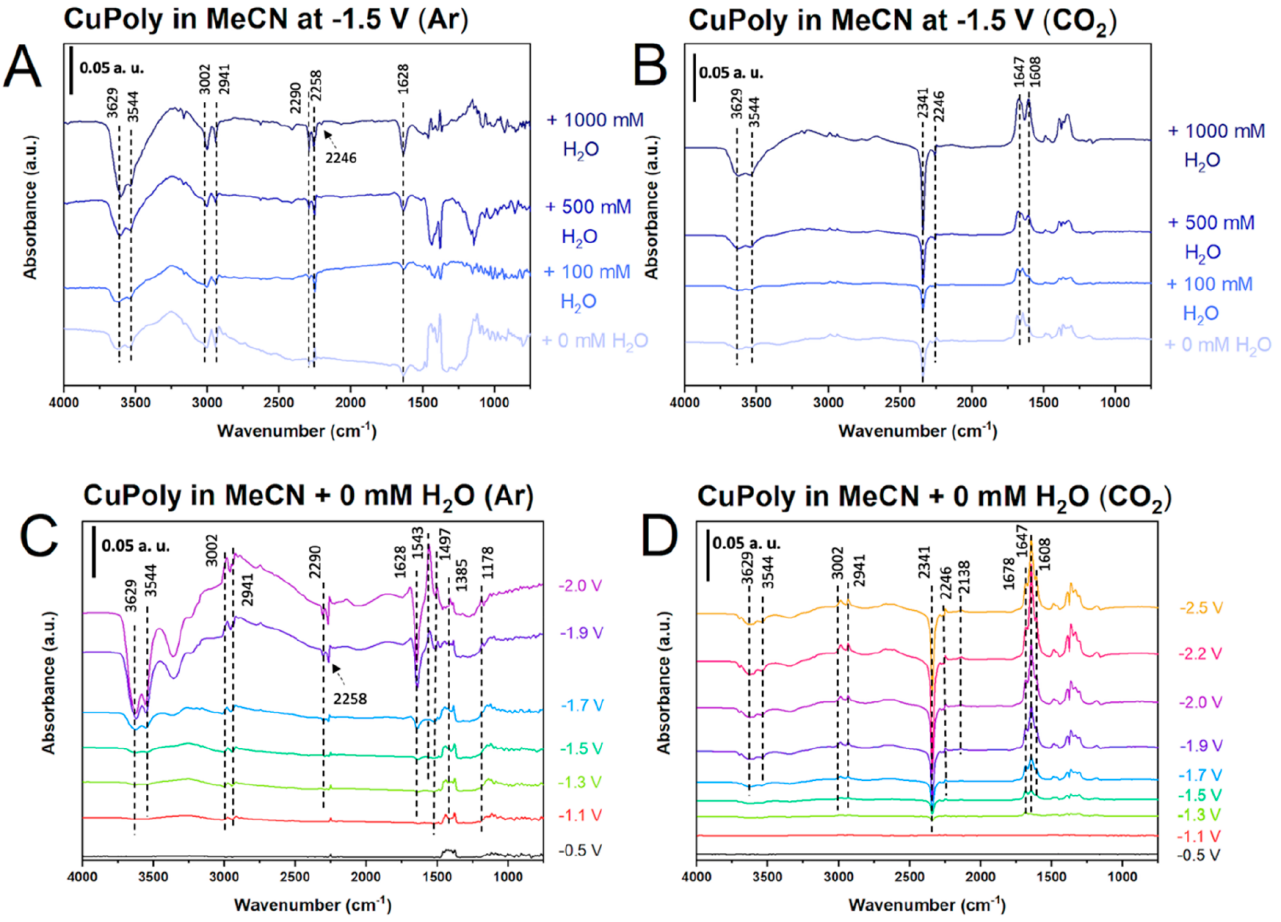
FTIR spectra for hydrogen evolution (A, C) and CO_2_ reduction (B, D) at CuPoly electrode in 0.1 M TEACl in CH_3_CN at the indicated water content and applied potentials. Reference spectrum was taken at −0.1 V vs Ag/Ag^+^.

The in situ FTIR spectra on CuPoly under an Ar atmosphere as a function of water content and applied potential are given in Figure 7A and C, respectively. Negative bands at 3629–3544 and 1628 cm^–1^, which are assigned to OH stretching and bending modes of residual water present in the acetonitrile solvent,^55^ respectively, become evident at potentials more negative than −1.5 V versus Ag/Ag^+^ (Figure 7C), in agreement with the cyclic voltammograms shown in Figure 4A. Their intensity also increases with increasing water concentration (Figure 7A), suggesting water is being reduced to produce hydrogen via 2H_2_O + 2e^–^ → H_2_ + 2OH^–^ reaction.

The spectra show common bands at around 3002–2941 cm^–1^, which are assigned to CH stretching from TEA^+^ and acetonitrile, respectively.^56^ In the C–N region of the spectra we observe three bands at 2290, 2258, and 2246 cm^–1^,^57^ which are less evident in the CO_2_ atmosphere. The bands at 2290 and 2246 cm^–1^ are assigned to the C–N stretching from acetonitrile free from interactions with anions,^21^ whereas the band at 2258 cm^–1^ is attributed to C–N groups from acetonitrile that interacts with chloride anion, similarly to what has been observed for BF_4_^–^ and ClO_4_^–^ groups at Cu and Pt electrodes, respectively.^21,58^

The positive bands in the region of 1543, 1497/1385, and 1178 cm^–1^ are assigned to C=O stretching, NH_2_ stretching, and NH_2_ bending modes, respectively, suggesting acetonitrile is decomposing to acetamide.^21^ These results agree with previous reports that found that, at more negative potentials, acetonitrile is decomposed into acetamide via a nucleophile attack of OH^–^ species generated during reduction of residual water^21,59^ We observed the related bands at slightly less negative potentials because before applying potentials for either HER or CO_2_RR, we performed a single voltametric scan in order to check the electrode surface in the spectro-electrochemical cell; however, this does not affect the interpretation of our data.

Additional bands are obtained when the electrolyte is saturated with CO_2_, as shown in Figure 7B,D. At −1.3 V (Figure 7D), the spectra show a negative band at 2341 cm^–1^ corresponding to CO_2_ consumption.^21^ This band becomes more intense with increasing amounts of water (Figure 7B) and at potentials more negative than −1.5 V (Figure 7D), which corresponds to the onset potential for CO_2_ reduction suggested by cyclic voltammetry (Figure 4B).

A high number of positive bands related to the formation of carbonyl species are observed in the wavenumber region around 1678–1608 cm^–1^ (Figure 7D).^21^ These bands increase as a function of potential at the same time as the negative band related to CO_2_ consumption increases, indicating those bands correspond to products from the CO_2_RR. More water in the system increases the production of the OH^–^ species due to water reduction, which in the presence of CO_2_ leads to the formation of carbonate and bicarbonate (HCO_3_^–^) species, as observed by the increase of the bands at 1608 cm^–1^, in agreement with previous literature.^21^ Once carbonate was not quantified as a final product, it justifies why the FE often does not reach 100% (Figure 6).

An interesting feature is revealed concerning the competition between the HER and the CO_2_RR in acetonitrile solvent. We found that the presence of CO_2_ in the acetonitrile electrolyte completely suppresses the band at 1628 cm^–1^, assigned to OH bending of water, while the bands at 3629–3544 cm^–1^ (OH stretching) are less intense than observed in the absence of CO_2_ (Figure 7A,C). These results confirm that in acetonitrile solvent, the presence of CO_2_ inhibits H_2_O accumulation at the surface and therefore its reduction to H_2_. We observe a small band at 2138 cm^–1^ (Figure 7D) that is assigned to the C–O stretching mode of CO gas, in agreement with the online gas chromatography analysis (Figure 6). In fact, in aprotic solvents, CO is formed as a product from CO_2_RR; however, due to its low solubility in acetonitrile, FTIR has a limited sensitivity for identifying CO.^21^ Unlike in aqueous solvent, where bridged and linearly adsorbed CO on Cu electrode were observed at 1800–1900 and 2050–2080 cm^–1^, respectively,^60^ our results do not show any band in this region of the spectra, confirming the CO-coverage on Cu electrode in acetonitrile is very low, making it difficult to be identified.

## 4 Discussion and Conclusion

Through a combination of cyclic voltammetry with online GC and in situ FTIR experiments, our results reveal that, similar to aqueous media,^27,37^ CO_2_RR selectivity toward C_2+_ products in acetonitrile is dependent on the structure and electrolyte composition. We found that CuNC is the only surface capable of producing 10% FE of ethylene if there is enough water present in the electrolyte (>500 mM). It appears that, in general, the qualitative effect of surface structure is the same in water and acetonitrile. Previous work done by Scholten et al.^29^ showed that flat electrodes favor HER over CO_2_RR and that only with the introduction of defects, as is the case of CuNC, the selectivity shifts toward C_2+_ hydrocarbons. Our results are also in accordance with previous work, which has shown that Cu(110) surfaces, which are more similar to copper polycrystalline surface, are more active for hydrogen evolution.^51^ This explains why CuPoly is more active for hydrogen evolution than CuNC in both electrolytes. Interestingly, whereas in aqueous media, CO_2_RR suppresses hydrogen evolution on both CuPoly and CuNC electrodes, the opposite is true in acetonitrile. In contrast to aqueous electrolytes, in acetonitrile solvent, the currents obtained for CuPoly and CuNC in a CO_2_-saturated electrolyte are higher than the currents obtained in an argon-purged electrolyte. Another remarkable difference is the quantitative selectivity toward CO, which is >85% on both CuPoly and CuNC in acetonitrile, whereas it is much lower in aqueous media on both CuPoly and CuNC (∼9.4% and 13.6%, respectively).

These results suggest that, in acetonitrile, CO desorbs much easier from the electrode surface than in aqueous media. The exact reason for this is not clear, but may be related to the stronger interaction of acetonitrile with the Cu, which then drives the CO off the surface. Remarkably, the addition of water to acetonitrile has little quantitative and almost no qualitative effect on the activity and selectivity of CO_2_RR. This strongly suggest that water is not strongly involved in rate-determining step of CO_2_RR in acetonitrile. Only at the highest water concentrations and at the CuNC surface, the CO coverage becomes high enough that a small amount of C_2+_ products are formed.

## Supporting Information Available

CVs of Fc/Fc^+^ in 0.1 M TEACl with 0 or 1 M of H_2_O, saturated with Ar or CO_2_; CVs of Cu nanospheres (CuNS) and Cu nanodendrites (CuND) electrodes in 0.1 M NaOH; Partial currents corresponding to various products recorded for the conditions reported in Figure 6; CVs of Cu nanocubes (CuNC) before and after electrolysis in 0.1 M NaOH (PDF)
