# Supplementary material for: Solvent Effect on Electrochemical CO2 Reduction Reaction on Nanostructured Copper Electrodes
Source: J Phys Chem C Nanomater Interfaces. 2023 Jul 12;127(29):14518–27. doi: 10.1021/acs.jpcc.3c03257 (PMC10388345; doi:10.1021/acs.jpcc.3c03257)
Supplement: Supplementary file 1 — jp3c03257_si_001.pdf [file jp3c03257_si_001.pdf]

Supporting Information:

Solvent Effect on Electrochemical CO_2 Reduction Reaction on Nanostructured Copper Electrodes

Connor Deacon-Price[1], Alisson H. M. da Silva[2], Cássia S. Santana[1], Marc T. M. Koper[2], Amanda C. Garcia[1,*]

1) Van't Hoff Institute for Molecular Sciences, University of Amsterdam, Science Park 904, 1098 XH, Amsterdam, the Netherlands

2) Leiden Institute of Chemistry, Leiden University, Gorlaeus Laboratories P.O. Box 9502, 2300 RA, Leiden, the Netherlands

*Corresponding author: a.c.garcia@uva.nl

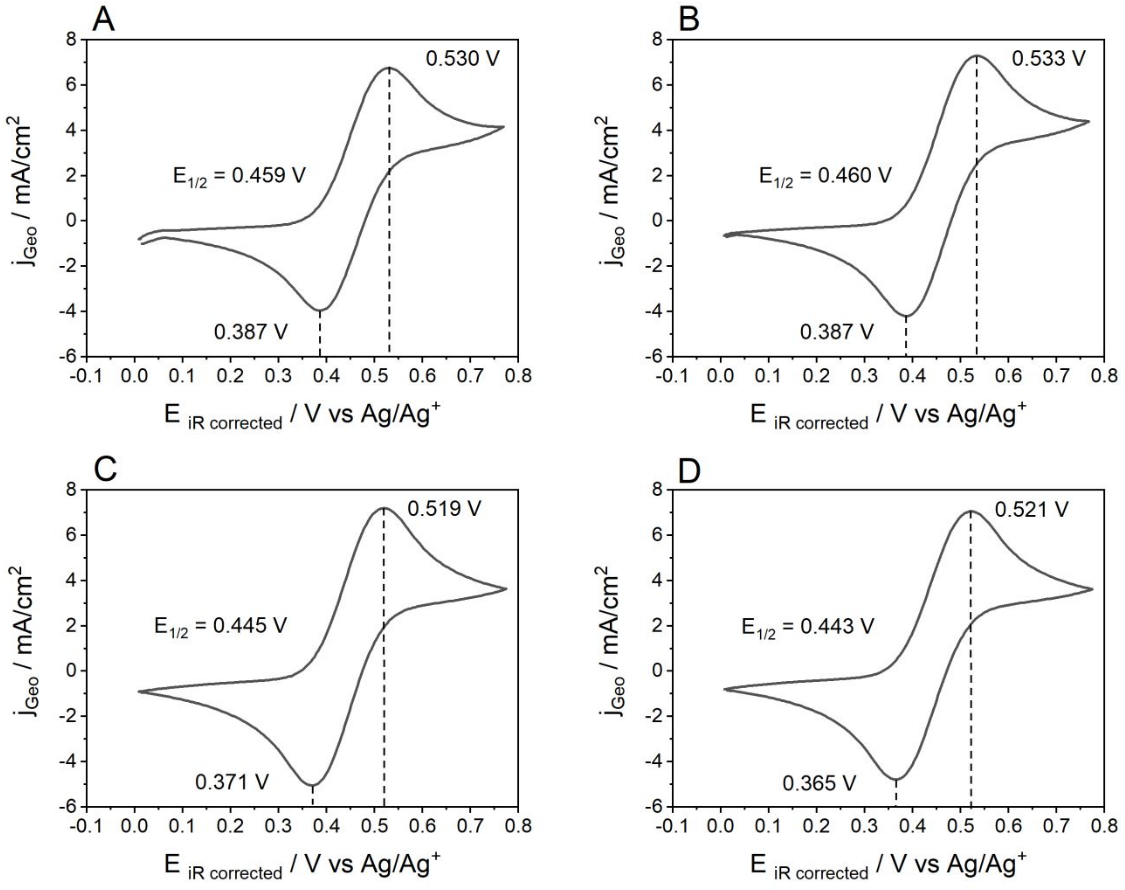

Figure S1. Cyclic voltammograms of Ferrocene/Ferrocenium (Fc/Fc^+) redox couple in 0.1 M TEACl with 0 M H_2O saturated with Ar (A); 0 M H_2O saturated with CO_2 (B); 1 M H_2O saturated with Ar (C); and 1 M H_2O saturated with CO_2 (D). Half wave potentials ($E_{1/2}$) are reported. WE = glassy carbon (redox couple not active over Cu); CE = Pt_{Poly}; RE = Ag/Ag$^+$. Scan rate of 50 mV s^{-1}.

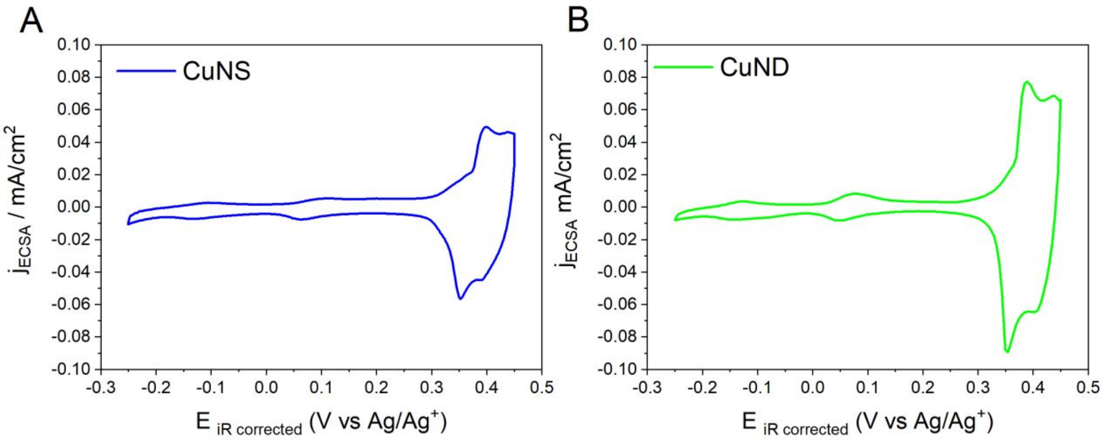

Figure S2. Cyclic voltammograms of Cu nanospheres (CuNS; A) and Cu nanodendrites (CuND; B) electrodes in 0.1 M NaOH, under Ar atmosphere, at a scan rate of 50 mV s^{-1}.

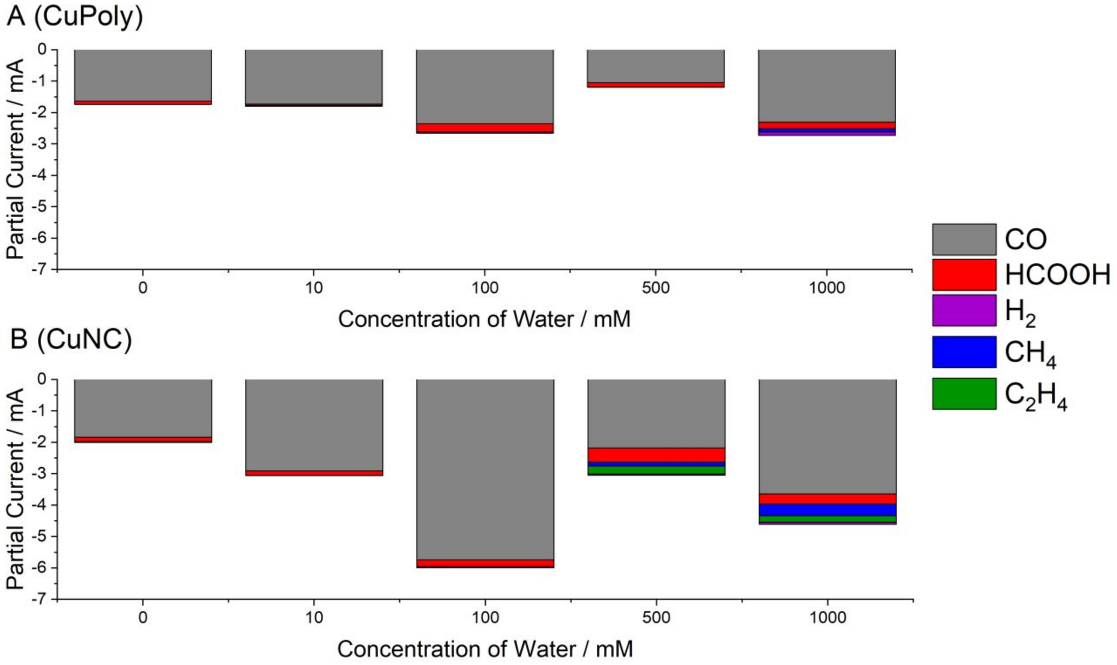

Figure S3. Partial currents corresponding to various products recorded for the conditions reported in manuscript (Fig. 6) used for FE calculation.

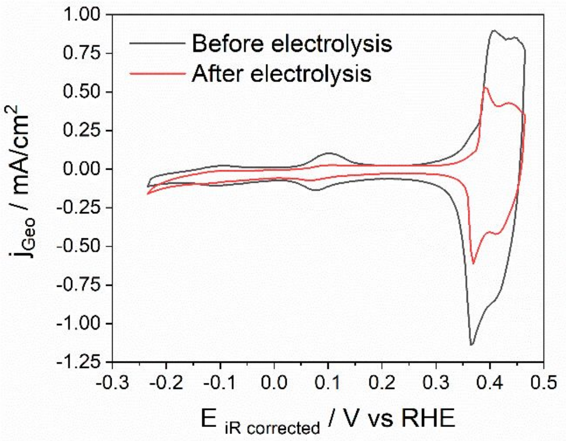

Figure S4. Cyclic voltammograms in 0.1 M NaOH, under Ar atmosphere, for CuNC before (black) and after (red) electrolysis performed at -2.0 V (vs Ag/Ag^{+}) for 90 minutes. Scan rate of 50 mV s^{-1}.
